# Dual roles of exostosin glycosyltransferase 1 in Zika virus infection

**DOI:** 10.1080/21505594.2025.2458681

**Published:** 2025-02-10

**Authors:** Jiaxin Ling, Asifa Khan, Matthias Denkewitz, Marco Maccarana, Åke Lundkvist, Jin-Ping Li, Jinlin Li

**Affiliations:** a Department of Medical Biochemistry and Microbiology, The Biomedical Center, Uppsala University, Uppsala, Sweden; b Zoonosis Science Center, Uppsala University, Uppsala, Sweden; c Department of Molecular Medicine, Biochemistry Unit, University of Pavia, Pavia, Italy; d Institute of Medical Virology, University Hospital Frankfurt, Goethe University,Germany; e SciLifeLab Uppsala, Uppsala University, Uppsala, Sweden

**Keywords:** ZIKV, EXT1, heparan sulfate, autophagy

## Abstract

Many factors involved in heparan sulfate (HS) biosynthesis and metabolism have been reported to play roles in viral infection. However, the detailed mechanisms are still not fully understood. In this study, we report that exostosin glycosyltransferase 1 (EXT1), the HS polymerase, is a critical regulatory factor for Zika virus (ZIKV) infection. Knocking out EXT1 dramatically restricts ZIKV infection, which is not due to the inhibition of virus entry resulting from HS deficiency, but mediated by the downregulation of autophagy. Induction of autophagy promotes ZIKV infection, and attenuated autophagy is found in distinct EXT1 knockout (EXT1-KO) cell lines. Induction of autophagy by rapamycin can relieve the ZIKV production defect in EXT1-KO cells. While over-expressing EXT1 results in the reduction of ZIKV production by targeting the viral envelope (E) protein and non-structural protein NS3 in a proteasome-dependent degradation manner. The different roles of EXT1 in ZIKV infection are further confirmed by the data that knocking down EXT1 at the early stage of ZIKV infection represses viral infection, whereas the increase of ZIKV infection is observed when knocking down EXT1 at the late stage of viral infection. This study discovers previously unrecognized intricate roles of EXT1 in ZIKV infection.

## Introduction

Zika virus (ZIKV) belongs to the Flaviviridae family [1], mainly including important human pathogens as dengue virus (DENV), Japanese encephalitis virus (JEV), West Nile virus (WNV), yellow fever virus (YFV), and Tick-borne encephalitis virus (TBEV). ZIKV is primarily transmitted by infected mosquitoes of *Aedes aegypti* and causes distinct symptoms ranging from asymptomatic, moderate (like fever and headache) to severe neurological disorders (microcephaly and Guillain-Barré syndrome) [2–4]. So far, there are no specific therapeutic treatments or vaccines available for ZIKV infection and associated diseases.

Like other flaviviruses, ZIKV is an enveloped, positive-sense and single-stranded RNA virus encoding 10 proteins including 3 structural proteins (capsid-C, precursor membrane-prM, and envelope-E) and 7 non-structural proteins (NS1, NS2A, NS2B, NS3, NS4A, NS4B, and NS5), which are the products from the cleavage of a polyprotein by viral and cellular proteases. The non-structural proteins are mainly responsible for the formation of a viral replication complex, virion assembly and evasion from host immune reactions [5–7]. Among the structural proteins, the E protein mediates the viral cell entry by interaction with cell receptors. In addition to the mutation in key amino acids, the post-modification of the E protein influences viral host cell entry and virulence. Glycosylation of the Asn (154) site on E may act as an attachment site for virus host entry [8]. Ubiquitination of the E protein drives ZIKV cell entry and pathogenesis [9]. Attenuation of the E protein ubiquitination by deubiquitinases has been reported to affect ZIKV infection [10,11].

Apart from viral functional receptors, heparan sulfate (HS) has been shown to be engaged in flavivirus cell entry as an attachment factor [12]. HS is a member of glycosaminoglycans (GAGs) family, which includes linear polysaccharides composed of repeating disaccharide units substituted by sulfate groups [13]. The biosynthesis of HS is precisely controlled by a series of enzymes. EXT1 and EXT2 are the two polymerases in HS biosynthesis responsible for the sequential transfer of sugar residues to the growing chain. Mutations in members of the EXT gene family, EXT1 or EXT2, have been associated with hereditary multiple osteochondromas [14,15]. The factors involved in HS biosynthesis have been suggested to play vital roles in flavivirus infections. By CRISPR screening, ten genes associated with HS biosynthesis were identified to have functions in JEV infection [16]. CRISPR/Cas9 screening indicated that NDST1 (N-deacetylase and N-sulfotransferase 1) and EXT1 might function as cell-dependent factors of ZIKV infection [17]. However, the functions of HS on ZIKV infection are controversial [12]. Recombinant ZIKV E protein can interact with HS by surface plasmon resonance [18]. The HS on the cell surface impacts ZIKV infection [19]. These studies supported HS as a dependency factor for ZIKV cell entry. In contrast, other studies showed that knocking out HS biosynthesis factors, downregulating HS, or removing cellular HS by enzymes exhibited no effect on ZIKV entry [20,21].

Autophagy is an evolutionally conserved cell process that degrades unnecessary components in a lysosome-dependent manner [22]. Autophagy has a complex relationship with virus infections, either by promoting or antagonizing virus replication [23]. Except for WNV [24], other members of the flaviviruses family utilize autophagy to facilitate viral replication (reviewed in [25]). An accumulating body of direct or indirect evidence suggests that ZIKV hijacks autophagy to support its replication in different cell and animal models [25–34], probably by utilizing the membrane structures associated with autophagic vesicles [25], though few studies pointed out an antiviral function of autophagy on ZIKV infection [35,36].

In this study, we utilized different EXT1 knockout cell models to study the roles of EXT1 in ZIKV infection. We found that ZIKV cell entry is not dependent on HS and the expression of EXT1 modulates ZIKV infection by regulating autophagy and viral envelope protein stability via a proteasome degradation way.

## Results

### EXT1 plays an essential role in ZIKV infection

**Figure 1.**
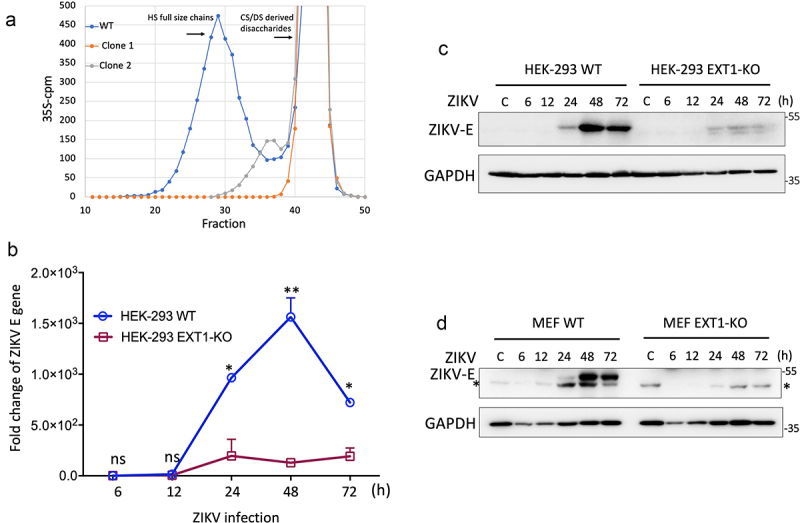
EXT1 is essential for ZIKV infection. (a) 35S-sulfate was added to the culture medium of the HEK293 EXT1-KO cell clones and HEK293 WT. The medium-derived in vivo labelled GAGs were purified. Labelled CS/DS was degraded by chondroitinase ABC and applied to size-permeation column superose 6. The position of full size HS chains was pointed out by an arrow in the figure. The prominent peaks eluting late in the chromatograms are derived from CS/DS degradation. (b) HEK-293 WT or HEK-293 EXT-KO cell lines were infected by ZIKV (MOI = 0.2). Cell pellets were harvested at different time points indicated in the figure. The replication of ZIKV was evaluated by qPCR using the specific primers targeting the ZIKV E gene. The results were presented as fold change relative to the infection of HEK-293 WT cell at 6 hpi and the data were shown as mean ± SD from two experiments. (c) The same samples collected from (b) were subjected to western blot by the antibody targeting the ZIKV E protein. The GAPDH was used as a loading control. (d) MEF WT or MEF EXT1-KO were infected by ZIKV (MOI = 0.2). Cells were collected at different time points post-infection, as indicated in the figure, and were prepared for western blot. The antibodies against the ZIKV E protein and GAPDH were used. The non-specific band detected by the anti-ZIKV E protein antibody in the MEF cells was indicated by an asterisk. Statistical analysis was performed using Student’s t-test. *p ≤ 0.05; **p ≤ 0.01, ns, not significant.

To dissect the roles of EXT1 in ZIKV infection, we first used the HEK-293 EXT1 knockout (KO) cells as the cell model. HEK-293 EXT1-KO cells were generated by CRISPR-Cas9. Due to the low expression of EXT1 in HEK-293 cells, we could not detect the endogenous EXT1 by western blot. Knocking out EXT1 will result in the absence of HS on the cell surface. Therefore, we tested if knocking out EXT1 was successful by measuring the expression of HS. The 35S labeled HS was analyzed by size filtration chromatography. In contrast to the full size and amount of HS chains produced by HEK-293 WT cells, only partial HS in both size and amount was found in the EXT1-KO clone 2 and almost no HS was detected in clone 1, suggesting EXT1 was successfully knocked out in clone 1 (Figure 1a). The clone 1 was used in the following experiments. Next, we monitored the ZIKV E protein expression at different time points post-infection. Compared to the HEK-293 WT cells, the mRNA expression of ZIKV E in HEK-293 EXT1-KO cells was significantly decreased from 24 hours post-infection (hpi) to the observation endpoint, 72 hpi, and no significant difference was observed at the early time points (6, and 12 hpi) (Figure 1b). The ZIKV E protein could only be detected at 24 hpi and obviously increased at 48 hpi. In line with the expression of mRNA, knocking out EXT1 markedly downregulated the ZIKV E protein expression at all the time points (Figure 1c). To rule out the possible off-target effects from CRISPR-Cas9 on ZIKV infection, we employed mouse embryonic fibroblast (MEF) EXT1-KO cells as another cell model to confirm the finding. MEF EXT1-KO cells were established from embryonic fibroblasts obtained from EXT1 mutant mice generated by the gene trap method [37]. In agreement with the results from HEK-293, the ZIKV E protein expression in MEF EXT1-KO cells exhibited a remarkable downregulation compared to the MEF-WT cells (Figure 1d).

### The cell entry of ZIKV is independent of HS on the cell surface

**Figure 2.**
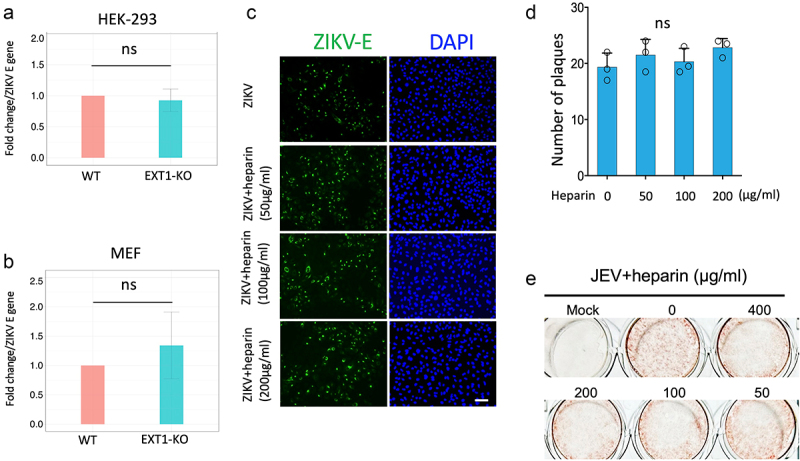
Heparan sulfate does not affect ZIKV cell entry. HEK-293 WT or HEK-293 EXT1-KO (a) and MEF-WT or MEF-EXT1-KO (b) were infected by ZIKV for 1.5 h. Cells were intensively washed and the amount of virus that entered the cells was quantified by qPCR using the specific primers targeting the ZIKV E gene. The results were shown as mean ± SD related to WT cell infection from three experiments. There is no significant difference of E gene between WT and EXT1-KO cells. (c) VeroE6 cells were infected by ZIKV (MOI = 0.5) for 20 h in the presence of different concentrations of heparin (50 µg/ml, 100 µg/ml, and 200 µg/ml). Cells were fixed and stained by the specific antibody against the ZIKV E protein and observed under microscopy. Representative micrographs of cells at different concentrations of heparin post ZIKV infection are shown. Scale bar = 50 μm. (d) An amount of ZIKV virus (which could form 20–30 plaques in one well of a 24-well plate) was incubated with different concentrations of heparin as indicated in the figure before infecting monolayer Vero E6 cells and the standard plaque assay was performed. The number of plaques in different conditions was counted and shown as mean ± SD from three experiments. There is no significant difference of plaque numbers in the presence of heparin compared to ZIKV infection alone. (e) The JEV was incubated with different doses of heparin and then used for infection of Vero E6 cells and followed by plaque assay. Statistical analysis was performed using Student’s t-test. ns, not significant.

EXT1 is a key enzyme in the chain elongation step of HS biosynthesis. HS has been reported as an attachment factor for the cell entry of many viruses, while the role of HS in ZIKV cell entry is still under debate (reviewed in [12]). Here, we aimed to investigate if the inhibition of ZIKV infection in EXT1 knockout cells was due to the blockage of viral entry caused by HS deficiency on the cell surface. MEF WT and MEF EXT1-KO or HEK-293 WT and HEK-293 EXT1-KO cells were infected by ZIKV and incubated at 37°C for 1 hour to enable virus entry. Then, the cells were subjected to intensive wash and ZIKV that entered the cells were assessed by quantitative PCR using specific primers targeting the ZIKV E gene. As shown in Figure 2(a,b), no significant difference in E gene expression was noticed between WT and EXT1-KO versions from both MEF and HEK-293 cells, suggesting that EXT1 deficiency did not affect ZIKV cell entry.

The function of HS on viral attachment is thought to be mainly mediated by the electronic interaction between negative-charged HS and positive-charged viral envelope proteins. Heparin, a clinically used anticoagulant, has a similar structure as HS but has more net negative charges compared to HS. Therefore, heparin is usually used as an inhibitor to evaluate if viral infection is dependent on HS. Here, we infected cells by ZIKV in the presence of different concentrations of heparin for 1.5 h and assessed the ZIKV infection by staining the ZIKV E protein at 20 hpi. No obvious difference of E-positive cells was observed among various concentrations of the heparin treatment (Figure 2c). We also utilized plaque assay to examine the effects of heparin on ZIKV cell entry. In accordance with the results from staining, the number of plaques in the treatment of heparin did not show a significant difference compared to that in ZIKV infection alone (Figure 2d). In contrast, the cell entry of JEV was dose-dependently inhibited by heparin (Figure 2e). All the data suggest that ZIKV does not use HS to assist its cell entry and knockout of EXT1 resulted in the inhibition of ZIKV infection is not because of the deficiency of HS on the cell surface.

### Loss of EXT1function downregulates autophagy, which attenuates ZIKV infection

**Figure 3.**
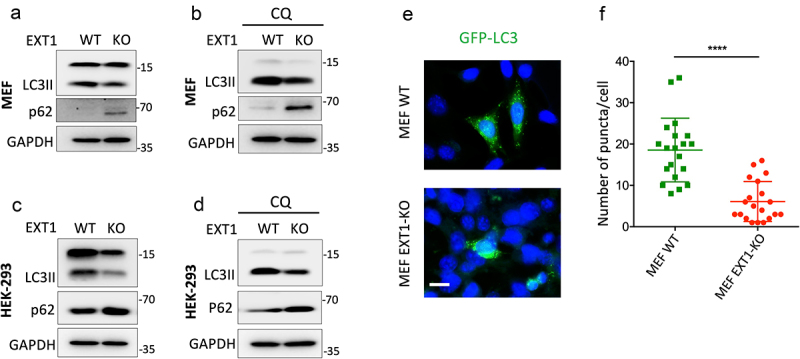
EXT1 knockout downregulates autophagy. MEF WT or MEF EXT1-KO and HEK-293 WT or HEK-293 EXT1-KO cells were seeded into plates and grown to 60–70% confluence. The cells were collected for detecting the expression of LC3II and p62. (a and c), or the cells were treated with chloroquine(CQ,10 µM) for another 6 h and were prepared for western blot (b and d). (e) The plasmid GFP-LC3 was transfected into MEF WT or MEF EXT1-KO cells. After 24 h, the cells were treated with CQ (20 µM) for 6 h, followed by fixation and observation under fluorescence microscopy. Representative micrographs acquired from MEF WT or MEF EXT1-KO are shown. Scale bar = 10 μm. (f) The number of GFP-LC3II puncta in MEF WT or MEF EXT1-KO. The Mean ± SD of the number of puncta in at least 20 GPF-LC3 positive cells recorded in each condition is shown. Statistical analysis was performed using Student’s t-test. ****p ≤ 0.0001.

Autophagy is closely engaged in ZIKV infection. HS has been reported to be implicated in autophagy regulation in Drosophila [38]. This information stimulated us to hypothesize that the deregulation of autophagy caused by EXT1 knockout may be responsible for the restriction of ZIKV infection. To test this hypothesis, we first investigated the effects of EXT1 knockout on autophagy by assessing the expression of a standard marker for autophagosomes, LC3II and an autophagosome cargo protein, p62/SQSTM1. In both MEF and HEK-293 EXT1-KO cells, downregulation of LC3II coupled with upregulation of p62 was detected (Figure 3(a,c)), which was not prevented by chloroquine (CQ), an autophagy inhibitor (Figure 3(b,d)). Reintroduction of EXT1 to MEF-EXT1-KO cells could partially recover the expression of LC3II (**Figure S1a**). This result indicated that knocking out EXT1 downregulated the autophagy. This conclusion was further confirmed by the results from monitoring the autophagosomes. The plasmid GFP-LC3 was transfected to MEF-WT and MEF-EXT1-KO cells, respectively, and the cells were treated with CQ. Compared to the MEF-WT cells, the GFP-LC3 puncta in MEF EXT1-KO cells were dramatically decreased (Figure 3(e,f)).

**Figure 4.**
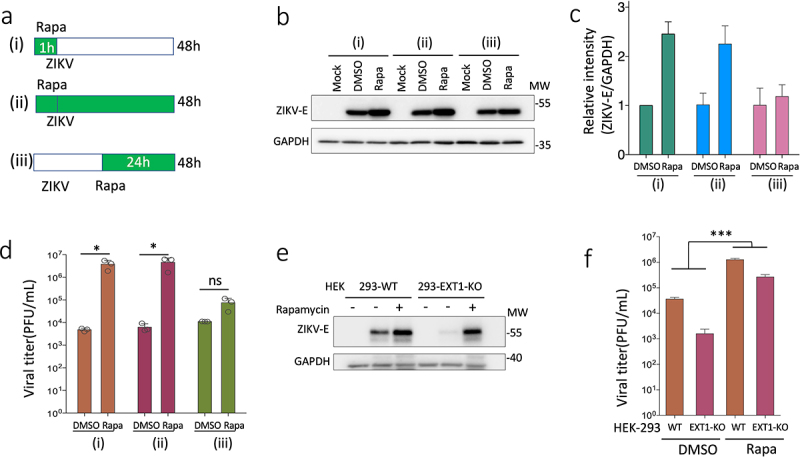
Autophagy promotes ZIKV replication. (a) The illustration of the rapamycin treatments at different stages of ZIKV infection. (i) HEK-293 cells were treated with rapamycin (Rapa, 1 µM) for 1 h followed by washing away and infecting the cells by ZIKV (MOI = 0.2) and continued to culture for a total of 48 h until the harvest of the cells. (ii) HEK-293 cells were treated with rapamycin (1 µM) for 1 h. The cells were infected by ZIKV and continued to culture in the presence of rapamycin for 48 h in total. (iii) HEK-293 cells were infected by ZIKV and rapamycin was added into the infected cells at 24 h post infection. The cells were continued to culture for another 24 h. In the three conditions, cells were also infected without adding rapamycin but adding the same amount of DMSO (since rapamycin was dissolved in DMSO) and cells were not infected (as mock infection). (b) The cell samples were collected in different conditions illustrated in (a) and cell lysates were prepared for western blot. The membrane was blotted using specific antibodies targeting ZIKV E protein and GAPDH separately. The representative blots from three experiments were shown. (c) For quantification, the intensity of ZIKV E specific bands in (b) was normalized to GAPDH. The data were shown as mean ± SD from three experiments. (d) The viral titres of supernatants from different experimental conditions in (b) were assessed by plaque assay. Statistical analysis was performed using Student’s t-test. *p ≤ 0.05; ns, not significant. (e) HEK-293 or HEK-293 EXT1-KO cells were infected by ZIKV with or without pre-treatment with rapamycin. The expression of the ZIKV E protein was examined by western blot. (f) The supernatants from various conditions in (e) were utilized to measure the viral titer. Statistical analysis was performed using two-way ANOVA. ***p ≤ 0.001.

Autophagy has been shown to have a proviral role in ZIKV infection in many studies [25–34]. To determine if autophagy favors ZIKV infection in our cell model, we used rapamycin to induce autophagy in the HEK-293 cells. In order to explore the possibilities that autophagy plays distinct roles in different phases of ZIKV infection, we treated the cells with rapamycin at different time points during ZIKV infection, as illustrated in Figure 4a. One-hour pretreatment of rapamycin followed by refreshing the media with or without rapamycin promoted the ZIKV E protein expression and virus production at a similar extent (Figure 4(b–d)), while adding rapamycin at 24 hpi, marginally changed viral protein expression and viral production (Figure 4(b–d)). These results suggest that the induction of autophagy by rapamycin enhances ZIKV infection in HEK-293 cells, which mainly happens at the early stage of ZIKV infection. Next, we aimed to check if the enhancement of autophagy could restore ZIKV infection in HEK-293 EXT1-KO cells. HEK-293 WT and HEK-293 EXT1-KO cells were infected by ZIKV with or without rapamycin treatment. Rapamycin treatment can partially rescue the expression of E protein and viral production in the HEK-293 EXT1-KO cells (Figure 4(e,f)). Altogether, the results from our cell models suggest that knocking out EXT1 wanes the autophagy, which compromises the ZIKV infection.

### Over-expression of EXT1impairs ZIKV replication

**Figure 5.**
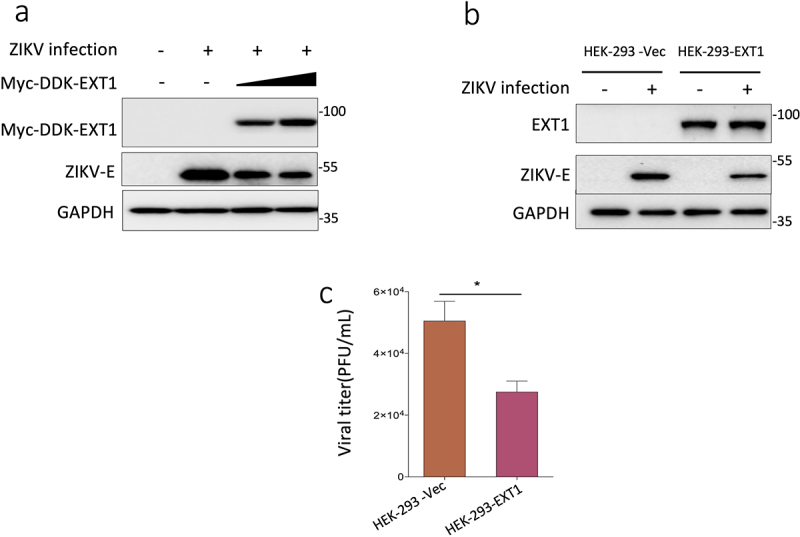
Overexpression of EXT1represses ZIKV replication. (a) Different amounts of Myc-ddk tagged EXT1(Myc-DDK-EXT1) plasmid were transfected into HEK-293 cells for 24 h followed by infection with ZIKV (MOI = 0.5). Cell pellets were harvested 48 h post-infection. The Myc-DDK-EXT1 and ZIKV E proteins expression were analyzed in western blots probed with the anti-FLAG and anti-ZIKV E antibodies separately. (b) HEK-293 cells stably expressing EXT1(HEK-293-EXT1) or HEK-293-empty vector cells (HEK-293-Vec) were infected with ZIKV (MOI = 0.5) for 48 h. The expression of the EXT1 and ZIKV E proteins were assessed by western blot using the anti-EXT1 and anti-ZIKV E antibodies. (c) The viral titers from the supernatants collected from (b) were analyzed using plaque assay. The data were shown as mean ± SD from two experiments. Statistical analysis was performed using Student’s t-test, *p ≤ 0.05.

Next, we aimed to examine if EXT1 acts as an essential factor that can boost ZIKV infection when it is over-expressed. Different amounts of plasmid encoding EXT1 were transfected into HEK-293 cells, followed by infection with ZIKV. The expression of ZIKV E protein was dose-dependently decreased with over-expression of EXT1 protein (Figure 5a). To rule out the possible effects of plasmid transient transfection on ZIKV infection, we constructed the HEK-293 cells stably expressing EXT1 (HEK-293-EXT1) and used it as a model to assess the impacts of EXT1 on ZIKV infection. As shown in Figure 5b,c, in line with the results from transient transfection, ZIKV infection was attenuated based on the evidence from the downregulation of E protein and the decrease of progeny virus production. Taking consideration of the compromised autophagy in EXT1-KO cells, we examined the LC3II marker in the HEK-293-EXT1 and HEK-293-Vec cells. There was no significant difference of LC3II expression in the HEK-293-EXT1 cells (Figure S1b). Altogether, the data indicate that upregulation of EXT1 hampers ZIKV infection.

### Over-expression of EXT1 results in ZIKV E and NS3 proteins degradation in a proteasome-dependent manner

In addition to the function as the regulator of HS to affect virus infection, EXT1 was reported to impede viral production by promoting viral protein degradation [39]. Therefore, we intended to test if EXT1 affects the viral protein stability in ZIKV infection in this set of experiments. EXT1 was co-transfected with the plasmid encoding one of ten proteins that are expressed by ZIKV (C, prM, E, NS1 NS2A, NS2B, NS3, NS4A, NS4B, and NS5) or an empty vector. Over-expression of EXT1 obviously downregulated the ZIKV E protein and NS3 but not other ZIKV proteins (Figure S2).

**Figure 6.**
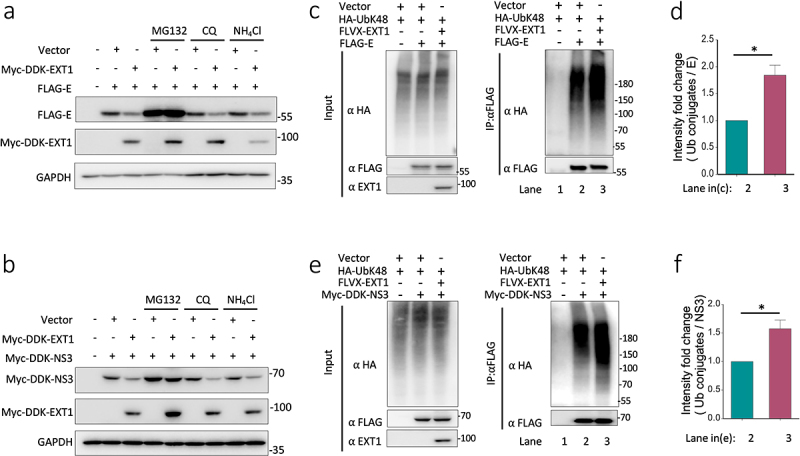
Overexpression of EXT1 leads to ZIKV envelope protein and NS3 protein degradation in a proteasome-dependent way. (a) FLAG tagged ZIKV E, or (b) myc-DDK tagged NS3 (myc-DDK-NS3) and myc-DDK tagged EXT1 (myc-DDK-EXT1) were co-transfected into HEK-293 cells in the presence or absence of proteasome inhibitor (MG132, 10 µM), and autophagy inhibitors (CQ = 15 µM, NH_4_Cl = 10 mM). The expression of proteins was analyzed in western blots probed with anti-FLAG antibody. (c, e). HA-UbK48 (HA-tagged Ub with only K48, other lysines mutated to arginines) was co-transfected with FLAG-E (c) or myc-DDK-NS3 (e) with untagged EXT1, FLVX-EXT1. Cells were treated with MG132 (10 µM) for 6 h before the immunoprecipitation with anti-FLAG beads in the denature conditions and the blots were probed with an anti-HA antibody. (d) The intensity of UbK48 conjugates in (c), and (f) the intensity of UbK48 conjugates in (e) were normalized by the intensity of the ZIKV E specific bands separately. The results were presented as mean ± SD from two experiments. Statistical analysis was performed using Student’s t-test. *p ≤ 0.05.

To further clarify the degradation pathway by which EXT1 mediated ZIKV E and NS3 protein degradation, we used a proteasome inhibitor, MG132, and two autophagy inhibitors, chloroquine (CQ) and ammonium chloride (NH_4_Cl), to treat the cells that co-transfected with EXT1 and FLAG-E or Myc-DDK-NS3. As shown in Figure 6a,b, the proteasome inhibitor but not the autophagy inhibitors prevented the degradation of FLAG-E and Myc-DDK-NS3, suggesting that EXT1 mediated ZIKV E and NS3 protein degradation in a proteasome-dependent manner.

Proteasomal degradation of protein is usually ubiquitination dependent. Several studies have demonstrated that the ZIKV E protein and NS3 could be ubiquitinated [9–11,40,41]. In a canonical function, K48-linked ubiquitination mediates the substrates for proteasome degradation, while K63-linked ubiquitin chains regulate critical biological signalling pathways including autophagy, DNA damage repair and immune response [42]. Co-transfection of HA-UbK48 (HA-tagged Ub with only K48, other lysines mutated to arginines) with FLAG-E or Myc-DDK-NS3 in the presence or absence of the plasmid expressing EXT1. EXT1 significantly boosted the K48-linked ubiquitination of the ZIKV E and NS3 protein (Figure 6(c–f)). All data indicate that EXT1 promotes proteasome-dependent degradation of the ZIKV E and NS3 protein by upregulating the K48-linked ubiquitination.

### The distinct roles of EXT1in the early and late stages of ZIKV infection

**Figure 7.**
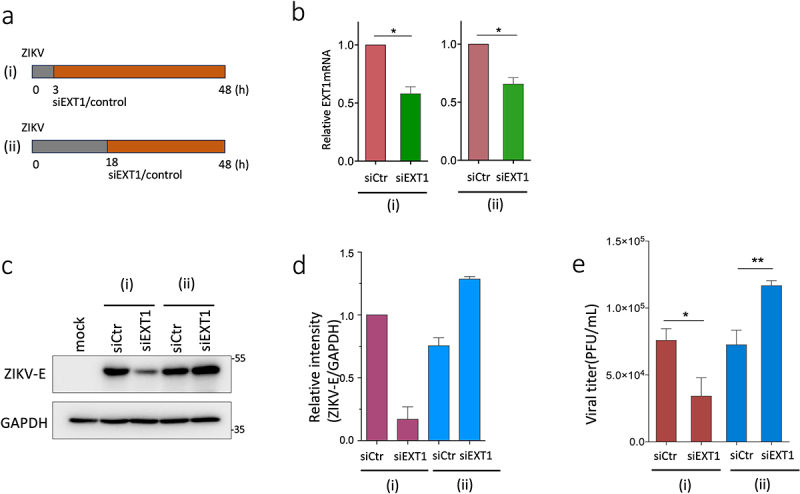
EXT1 plays distinct roles in the different stages of ZIKV infection. (a) The illustration of the knocking down EXT1 at different stages of ZIKV infection. (i) HEK-293 cells were infected by ZIKV (MOI = 0.2). After 3 h, the infected cells were transfected with siEXT1 (50 nM) or control siRNA (siCtr, 50 nM); (ii) HEK-293 cells were infected by ZIKV. After 18 h, the infected cells were transfected with siEXT1 or siCtr at a concentration of 50 nM. The cells were cultured for 48 h in total until harvest. (b) The expression of EXT1mRNA at different conditions as illustrated in (a). (c) The expression of ZIKV E protein was assessed by western blot from the different conditions in (a). (d) For quantification, the intensity of ZIKV E specific bands in (c) was normalized to GAPDH. The data were shown as mean ± SD from two experiments. (e) The viral titers of supernatants from cells treated with different experimental conditions in (c) were assessed by plaque assay. The results were presented as mean ± SD from two experiments. Statistical analysis was performed using Student’s t-test. *p ≤ 0.05; **p ≤ 0.01.

The results from ZIKV infection in EXT1-KO and EXT1 over-expression cell model suggest the intricate functions of EXT1 during the ZIKV infection. EXT1 is needed for the initiation of ZIKV replication at an early stage, while EXT1 will promote the degradation of NS3 and E, which will hinder the ZIKV replication at the subsequent stages. To further confirm this hypothesis, we knocked down EXT1 expression at different time points post ZIKV infection, as illustrated in Figure 7a. Both 3 hpi and 18 hpi knocking down resulted in 40–50% downregulation of EXT1 mRNA expression (Figure 7b). The 3 hpi knocking down EXT1 significantly downregulated the ZIKV E protein expression, whereas 18 hpi knocking out upregulated the expression of the ZIKV E protein (Figure 7(c,d)). In agreement with the effects on the viral protein expression, the production of ZIKV was waned and boosted when EXT1 was knocked down at 3 hpi and 18 hpi, separately (Figure 7e). Collectively, these findings corroborated the conclusion that EXT1 plays dual roles in ZIKV infection at the different stages of infection.

## Discussion

Cumulative studies discovered the functions of enzymes for HS biosynthesis and metabolism in viral infectious diseases. Here, we showed that EXT1 plays distinct roles in ZIKV infection by remodeling autophagy and regulating viral protein stability.

The roles of HS in ZIKV infection are still under debate. Several pieces of evidence suggest that ZIKV cell entry is HS-dependent. Low pH (pH = 6.2) promoted ZIKV infection by increasing the binding of viral particles to HS on the cell surface [19]. Another study, however, pointed out that ZIKV infectivity was higher after adjusting to a pH of 9 before infection [43]. Heparin or Heparan sulfate was shown to bind to the recombinant ZIKV E protein using surface plasmon resonance [18]. In contrast, heparin turned out to promote ZIKV infection in the Vero cell model [44]. More direct evidence comes from the cell model where the HS biosynthesis enzymes were knocked out and the results indicated HS deficiency did not affect the entry of ZIKV [20]. The discrepancies in findings regarding the role of HS in ZIKV infection may stem from differences in cell types and experimental methodologies employed across studies. Furthermore, these inconsistencies may be linked to the intricate nature of the interaction between ZIKV and HS.

Using different approaches and EXT1 knockout cell models, we demonstrated that HS on the cell surface was not involved in ZIKV cell entry, which is in line with other evidence [20]. This feature of ZIKV seems to be different from other members of the flaviviruses, such as DENV and JEV, which utilize HS as a co-attachment factor [45,46]. This is confirmed in our study by the data that JEV entry was inhibited in the presence of heparin. The assistant role of HS for virus cell entry is thought to be mainly due to the interaction of positive-charged viral glycoprotein and negative-charged HS. Heparin could not impede ZIKV cell entry, suggesting the electrostatic interaction of HS and ZIKV E protein does not affect viral binding to the main receptors. This may be due to the electrostatic interaction itself being relatively weak or that other factors play a dominant role in mediating the binding of ZIKV E protein to the receptors. It is worth noting that the ZIKV E protein consists of a more compact surface on the viral particle surface as compared to DENV [8,47]. Additionally, ZIKV entry into cells independent of HS provides an optimal model to investigate the potential functions of factors involved in HS biosynthesis during the infection process of positive-stranded RNA viruses after cell entry.

EXT1 forms a hetero-oligomeric complex with EXT2 and acts as the HS polymerase. A direct consequence of EXT1 knockout is the loss of HS on the cell surface. In our study, knocking out EXT1 in different cell models resulted in a decrease in autophagy. The disruption of HS biosynthesis may account for the downregulation of autophagy since HS functions as a co-receptor for plenty of signaling molecules. However, in the drosophila model, the knock-down of the *ttv* gene (a homolog of EXT1 in mammalian cells) by RNA interference technology upregulated autophagy in muscle cells and fat body cells [38]. The biological significance of different regulations of EXT1 on autophagy between mammalian and drosophila cells and whether this difference is cell-type dependent are interesting questions that need to be elucidated in future studies. Actually, EXT1 knockout will substantially remodel endoplasmic reticulum (ER) and Golgi including the changes in the molecular composition of ER membranes and secretory cargo trafficking [48]. EXT1down-regulation also induces Golgi reorganization and a metabolic switch [48]. The ER – Golgi intermediate compartment is thought to be the membrane resource for initiating the formation of autophagosomes [49]. So, it will be not surprising that the alteration of ER and Golgi caused by EXT1 knocking out compromises autophagy.

Flavivirus and many other positive-stranded RNA viruses have been shown to be dependent on the initiation of the autophagic pathway for their effective replication [50]. Autophagy plays multifaceted roles in ZIKV infection. More direct evidence suggests that ZIKV induces autophagy to promote viral replication [25,27,28,31,33,34], while inhibits the selective autophagy targeting viral proteins [34,51], and hijacks autophagosome-derived secretory organelles to release progeny virus from host cells [52]. The solid evidence supporting the induction of autophagy in favor of ZIKV infection comes from the colocalization of cytosolic microtubule-associated molecule LC3 and the ZIKV E protein in the infected fibroblasts as well as the observation of elevated ZIKV replication when stimulates the autophagosome formation, which suggests ZIKV utilizes the autophagic vesicles as viral replication site [25]. In contrast to human fetal neural stem cells, neural stem cells, and mouse models [27,34], in the ZIKV-infected Drosophila brain, inflammation-induced autophagy hampers viral infection [53]. In our study, adding rapamycin to induce autophagy by inhibiting mTOR boosted ZIKV replication, which is in accord with previous studies that showed rapamycin promoted ZIKV replication [27,28,34]. Since different cell models were used in our and other studies, it suggests that enhancement of ZIKV infection by induction of autophagy using rapamycin is cell type independent. Besides, our study indicated that it is sufficient to promote ZIKV replication by treatment of cells with rapamycin at the early time point of viral infection, which agrees with the previous hypothesis that ZIKV subverts autophagosome for viral replication at the early infection stage [25]. Enhancement of autophagy promotes ZIKV replication, which was further supported by our data that ZIKV replication was restrained in EXT1KO cells, whose autophagy was discovered to be compromised.

In addition to the key role of EXT1 in the initiation of ZIKV replication by remodeling autophagy, we also found EXT1 could promote K48-linked polyubiquitination of ZIKV E and NS3 proteins and result in the subsequent proteasome-dependent degradation. In another positive-strand RNA virus, porcine reproductive and respiratory syndrome virus (PRRSV) infection, EXT1 was reported to promote the K48-linked polyubiquitination of PRRSV Nsp3 and Nsp5 [39]. Whether in other flaviviruses or even in positive-strand RNA virus infection, EXT1 also engages in the regulation of viral protein stability by a proteasome-dependent pathway remains an extremely interesting question.

**Figure 8.**
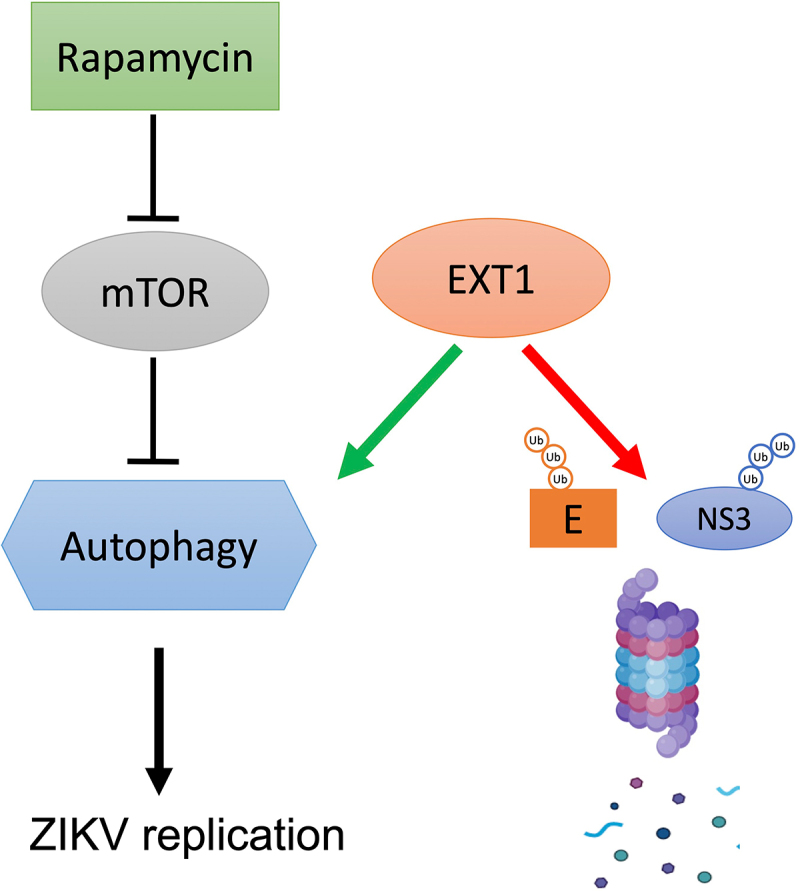
The proposed working model of EXT1’s intricate roles in ZIKV infection. ZIKV depends on the initiation of the autophagic pathway for its effective replication. Induction of autophagy by rapamycin, especially at early stages of infection, escalates ZIKV replication. EXT1is essential to sustain the autophagy level, which could be utilized by ZIKV to initiate its replication effectively, suggesting EXT1 is a proviral factor. Additionally, EXT1can promote the degradation of ZIKV E and NS3 proteins in a proteasome-dependent way, pointing out EXT1as a restriction factor against ZIKV. The proteasome icon is from BioRender.com.

In this study, we uncovered the intricate roles of EXT1 during different stages of ZIKV infection (Figure 8). ZIKV’s replication is dependent on the initiation of the autophagy pathway and induction of autophagy will enhance ZIKV replication. Knocking out EXT1 results in the downregulation of autophagy, which will lead to the restriction of ZIKV replication. In this sense, EXT1 sustains the autophagy level, which would be utilized by ZIKV to initiate its replication effectively. Additionally, EXT1 can target ZIKV E and NS3 proteins by stimulating K48-linked polyubiquitination and subsequent degradation, pointing out EXT1 as a restriction factor against ZIKV (Figure 8). However, our current study only scratches the surface of EXT1’s multifaceted roles in virus infection. Many interesting questions associated with this study are worth to investigate in the future. For example, (I) How does EXT1 remodel autophagy?, (II) As a key regulator of ER morphology and dynamic, whether EXT1 selectively targeting exogenous viral proteins is a conserved mechanism to keep the homoeostasis of ER and (III) whether the interaction of EXT1 and EXT2 impacts the EXT1’s roles in viral infection? Altogether, our study provides a new understanding of how EXT1 modulates ZIKV infection in different ways and raises interesting questions about EXT1’s roles in virus infection.

## Material and methods

### Antibodies and chemicals

The following chemicals and antibodies were used in this study. Chemicals: rapamycin (Sigma-Aldrich 553,210), MG132 (MedChemExpress, HY-13259), Chloroquine (InvivoGen, 50-63-5), heparin (Hepalink, Inj010702, Shenzhen); Antibodies: LC3B Antibody (Cell signaling, 2775), SQSTM1/P62 antibody [N3C1] (Gene Tex, GTX100685), Zika virus Envelope protein antibody (Gene Tex, GTX133314), Japanese encephalitis virus NS3 antibody (Gene Tex, GTX125868), GAPDH Monoclonal antibody (Proteintech 60,004–1-Ig), Monoclonal ANTI-FLAG® M2 antibody (Sigma, F3165), ANTI-FLAG® M2 Affinity Gel (Sigma, A2220), EXT1 Antibody (A-7) (Santa Cruz, sc -515,144), HA-Tag Antibody (F-7) (Santa Cruz, sc-7392), Purified anti-V5-tag Antibody (Biolegend 680,601). Goat anti-Mouse IgG (H+L) Secondary Antibody, HRP (Invitrogen 31,430), Goat anti-Rabbit IgG (H+L) Cross-Adsorbed Secondary Antibody, HRP (Invitrogen, G21234), Goat anti-Rabbit IgG (H+L) Cross-Adsorbed Secondary Antibody, Alexa Fluor™ 594 (Invitrogen, A-11012).

### Plasmids and siRNA

FLAG tagged ZIKV gene expression plasmids were generated by amplification of the CDS using cDNA from the strain ZIKV MR766 as the template and cloned between the PstI and XhoI restriction sites in pCMV-3Tag plasmid (for ZIKV E and NS1) and between the SgfI and MluI restriction sites in pCMV6-Entry plasmid (for ZIKV Capsid, pr-Membrane, NS2A, NS2B, and NS3). pcDNA3.2/NS4A(Addgene#1208826), pcDNA3.2/NS4B(Addgene#120823), pcDNA3.2/NS5(Addgene#120825) were gifts from Jan Rehwinkel [54]. Myc-DDK-tagged EXT1 (Origene, CAT#: RC200644) was kindly provided as a gift by Prof. Marion Kusche Gullberg from the Department of Biomedicine, University of Bergen, Norway. pSELECT-GFP-hLC3 was purchased from InvivoGen (France, psetz-gfplc3). The CDS of EXT1was amplified by specific primers (5´-ATGCAGGCCAAAAAACGCTATT-3´ and 5´-AAGTCGCTCAATGTCTCGGTATT-3´) and cloned in the Xhol and Xbal sites of PLVX-IRES-Puro Vector(Clontech, TaKaRa) to construct FLVX-EXT1 for generating an EXT1 stably expressing cell line. HA-UbK48 was kindly provided by Prof. Carl-Henrik Heldin’s group from IMBIM, Uppsala University [55]. siGENOME Non-Targeting Control siRNAs (D-001210-03) and siGENOME Human EXT1 siRNA (*M*-011030-00) were purchased from Dharmacon Reagents.

### Cell lines and virus strains

HEK-293 (Human Embryonic Kidney cells, ATCC CRL-1573), MEF (Mouse embryonic fibroblasts, ATCC CRL-2991) and VeroE6 (ATTC CRL-1586) were cultured in Dulbecco’s minimal essential medium (DMEM, Gibco), supplemented with 10% FBS (Gibco) and 100 units/ml of penicillin and 100 μg/ml of streptomycin (Gibco), and grown in a 37°C incubator with 5% CO_2_. MEF EXT1-KO cells was a gift from Dr. Marion Kusche Gullberg from the Department of Biomedicine, University of Bergen, Norway. The HEK-293 EXT1-KO cell line was generated using CRISPR-Cas9 by cloning the specific guide sequence (5´-TGGGTCCTTCAGATTCCTGG-3´) to lentiCRISPRv2 backbone according to methods provided by Zhang Lab GeCKO website. HEK-293T cells were co-transfected with the plasmids psPAX2 and pMD2G (psPAX2 and pMD2G were a gift from Didier Trono Addgene plasmid #12260 and # 12259) and lentiCRISPRv2 with guide sequence to produce lentivirus. The lentivirus-infected HEK-293 cells were selected by 2 µg/ml puromycin for at least two weeks and several cells were selected as “clones” for further validation. EXT1 knockout cells were functionally confirmed by measuring the HS amount and chain length. To construct the HEK-293 cell stably expressing EXT1, lentivirus was packaged by co-transfection of FLVX-EXT1 with the 2nd generation lentiviral systems plasmids psPAX2 and pMD2G. Lentivirus-transduced HEK-293 cells were selected under 2 µg/ml puromycin for at least 2 weeks and the expression of EXT1 was examined by western blot using an antibody against EXT1. African ZIKV isolate (MR766) and JEV (Nakayama) were used in this study.

### HS analysis

The analysis was performed according to the previous study [56] with some modifications. Briefly, GAGs were *in vivo* labeled by adding 100 uCi/ml 35S-sulfate (Biotech-IgG Equity AB) in sulfate-poor labeling medium (Gibco cat. no. 074-91083P) when cells were 70–90% confluent in 6-well plates. After 24 hours of labeling, medium was collected, and GAGs were released from proteoglycan by beta-elimination (final 0.5 M NaOH, 0.1 M sodium borohydrate, 16 hours at 4°C). After acidification to destroy sodium borohydrate and subsequent neutralization, the released GAGs were purified by DEAE-Sephacel (ThermoFisher) anion exchange chromatography. Bound GAGs were washed with PBS, eluted in PBS containing 2 M NaCl, and desalted using PD-10 columns (ThermoFisher). Chondroitin/dermatan sulfate (CS/DS) were exhaustively depolymerized to disaccharides by incubation of the labeled GAGs with 10 mIU of chondroitinase ABC (Sigma; overnight incubation in 50 mm NH_4_OAc pH 8.0). The amount and chain length of HS was investigated by the size-permeation column Superose 6 (Thermofisher) run in 0.2 M NH_4_HCO_3_.

### Western blot and immunoprecipitation

The cells were washed once by cold PBS and lysed using RIPA buffer (25 mm Tris•HCl pH 7.6, 150 mm NaCl, 1% NP-40, 1% sodium deoxycholate, 0.1% SDS). The lysate was denatured at 100°C for 10 min in Laemmli sample buffer and fractionated in homemade SDS-PAGE gels or acrylamide Bis-Tris 4–15% gradient gels (Bio-Rad). After being transferred onto a PVDF membrane (Millipore), the membrane was subjected to blocking in TBS containing 0.1% Tween-20 and 5% non-fat milk. The membrane was incubated with a proper primary antibody for 1 hour at room temperature or overnight at 4°C, followed by a 1-hour incubation with a corresponding horseradish peroxidase-conjugated secondary antibodies. Visualization of the immunocomplexes was achieved by Super Signal West Pico PLUS substrate (ThermoFisher) using the ChemiDoc System (Bio-Rad). For the immunoprecipitation assay, the cells were lysed on ice for 30 min (50 mm Tris-HCl pH 7.4, 150 mm NaCl, 1% Triton X-100, 1 mm EDTA, 10% glycerol, 20 mm NEM, 2 mm Iodoacetamide, protease inhibitors cocktail). For immunoprecipitations under the denaturing condition the lysis buffer was supplemented with 1% SDS, followed by dilution to 0.1% SDS before IP. In order to precipitate FLAG tagged proteins, the lysate was incubated with 45 µl ANTI-FLAG® M2 Affinity Gel (A2220; Sigma) at 4°C for three hours with rotation followed by three-times washing by lysis buffer and one time washing by TBS (pH = 7.4) with protease inhibitors. The immunocomplexes were eluted using FLAG peptide (F4799; Sigma) by incubation at 4°C for 30 min.

### Quantitative PCR

Total RNA was extracted from cell samples using RNeasy Mini Kit (Qiagen 74,104) based on the protocol provided by the company. The concentration and quantity of RNA were evaluated by Nano drop-2000 (ThermoFisher). Around 1 µg RNA was used to perform the reverse transcription by High-Capacity Reverse transcription kit (Applied Biosystem 4,368,813) using the protocol: 25°C for 10 min; 37°C for 120 min; 85°C for 5 min. One µl of cDNA mixture was utilized for quantitative PCR by Power Track SYBR Green Master Mix (Applied Biosystems, A46109) with cycling conditions: 95°C for 30 sec, followed by 45 cycles of denaturation at 95°C for 10 sec and annealing/extension at 60°C for 60 sec. Melt curve analysis was added by running from 65°C to 95°C with 0.5°C increments at 5 sec/step. Fold change was calculated as: Fold Change = 2-Δ(ΔCt) where ΔCt = Ct _target_−Ct _housekeeping_ and Δ(ΔCT) = ΔCt _treated_− ΔCt _untreated_. The primers used in this study for quantitative PCR are listed below. ZIKV E gene (F-5´TTGGTCATGATACTGCTGATTGC3´; *R*-5´CCTTCCACAAAGTCCCTATTGC3´); human GAPDH gene (F-5´TGGGCTACACTGAGCACCAG3´; *R*-5´ GGGTGTCGCTGTTGAAGTC3´) and mouse GAPDH gene (F-5´ TGAAGCAGGCATCTGAGGG3´; *R*-5´CGAAGGTGGAAGAGTGGGAG3´).

### Immunofluorescence assay

Cells were seeded into a plate with round coverslips in the well. To perform immunofluorescence analysis, the cells were fixed by cold methanol for 20 min at −20°C. The methanol was removed and room temperature (RT) PBS was added to wash the cells 3 times × 5 min. The fixed cells were permeabilized by 0.05% TritonX-100 in PBS and blocked with 4% bovine serum albumin (BSA) for 40–60 min. The primary antibodies with a proper dilution in 4% BSA were utilized to incubate with cells at RT for 1 h followed by 4 times × 5 min washing with PBS. Then, appropriate Alexa Fluor-conjugated secondary antibodies were prepared and incubated with the cells at RT for 1 h in a dark incubation box, followed by 4 times washing with PBS. The coverslips were rinsed several times with distilled water before being put on the microscope slide with a mounting buffer containing DAPI (Vector Laboratories, Inc. Burlingame, CA, USA). The coverslips were sealed with nail polish. The images were acquired by a fluorescence microscope (Eclipse 90i, Nikon, Tokyo, Japan). Analysis of images was performed by using the ImageJ software. For checking GFP-LC3 puncta, MEF WT and MEF EXT1-KO cells were grown on coverslips in 12-well plates and transfected with GFP-LC3 plasmid using Lipofectamine 3000 (Invitrogen, L3000001). Cells were treated with 20 μM chloroquine or DMSO for 6 hours before fixation and observation under a fluorescence microscope.

### Plaque assay

VeroE6 cells were seeded into 24-well plates. When the cells were grown to monolayer, serial dilutions of the supernatant containing the infectious ZIKV (MR-766) were incubated with the cells at 37°C for 1 h by shaking the plate 3–4 times. The mixture was removed and followed by adding 1 ml 0.8% agar media (Sigma-Aldrich, A5431) to each well. After 72 h, cells were fixed and stained by 0.5% Crystal Violet Solution (Sigma-Aldrich, V5265) and the cells were washed with distilled water for several times. The number of plaques were counted. For the inhibition of heparin on ZIKV or JEV, the virus was incubated with heparin for 1 h at 37°C and then used for infection of Vero E6 cells. Regarding the plaque assay for JEV (Nakayama), instead of staining with crystal violet, the infected cells were fixed by 4% paraformaldehyde after 72 h and stained by anti-JEV NS3 antibody.

## Supplementary Material

Fig S1 R1.jpg

Fig S2 R1.jpg

Supporting_information - clean copy.docx

## Data Availability

The data that support the findings of this study are openly available in https://doi.org/10.6084/m9.figshare.26862613
