# Supplementary figures and images for: Dual roles of exostosin glycosyltransferase 1 in Zika virus infection
Source: Virulence. 2025 Feb 10;16(1):2458681. doi: 10.1080/21505594.2025.2458681 (PMC11812395; doi:10.1080/21505594.2025.2458681)

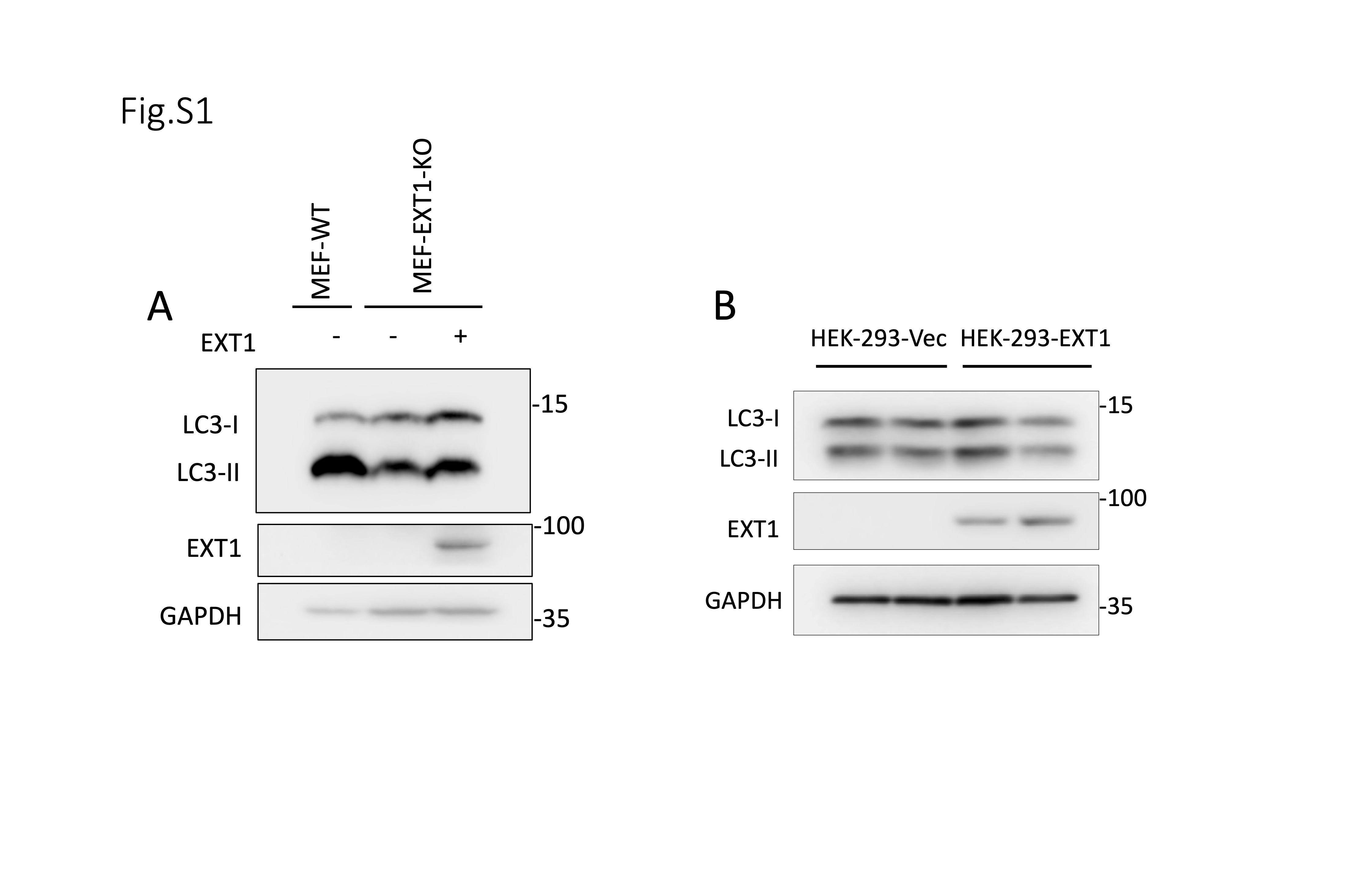

Supplement: Fig S1 R1.jpg [file KVIR_A_2458681_SM4361.jpg]

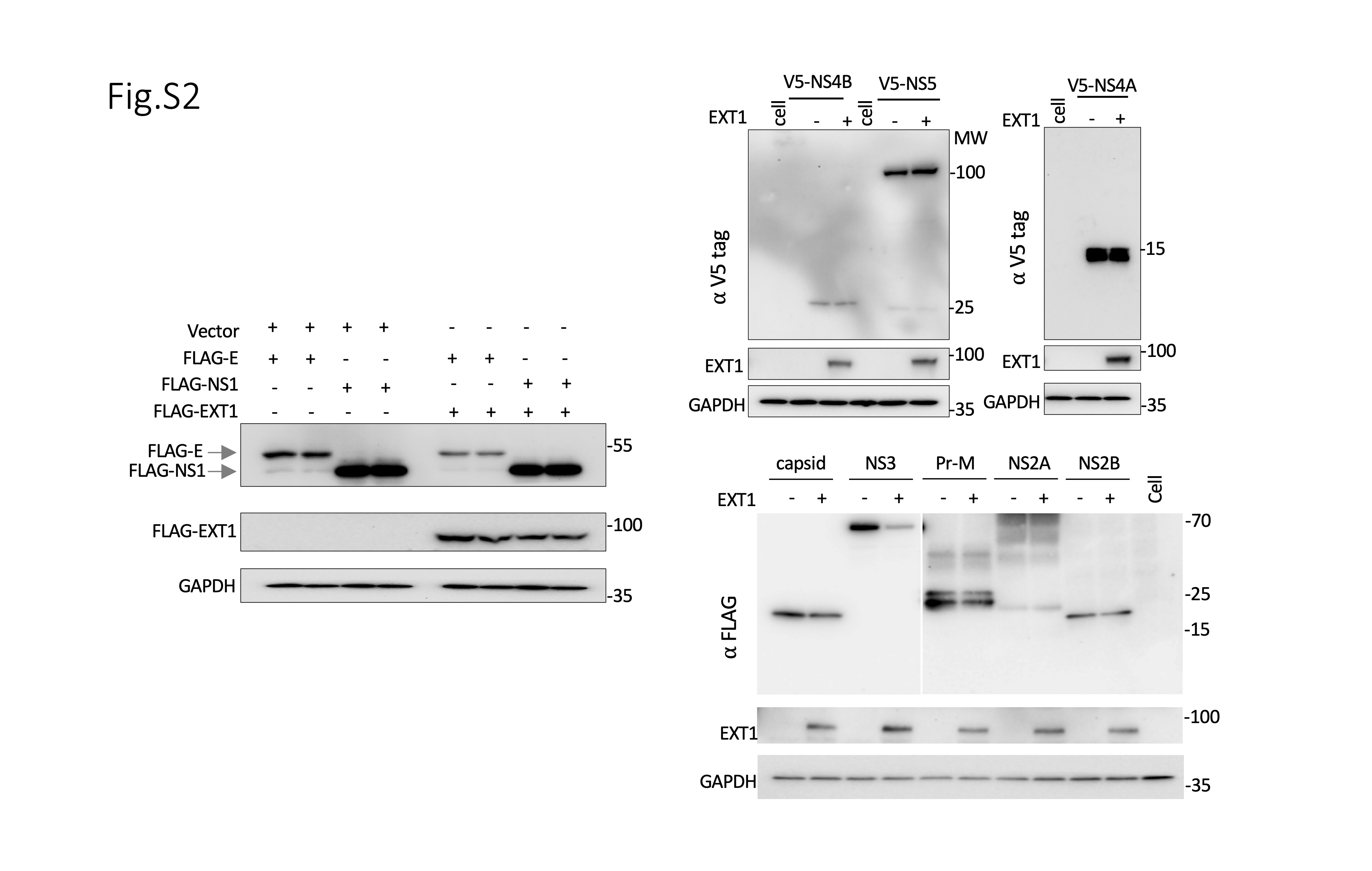

Supplement: Fig S2 R1.jpg [file KVIR_A_2458681_SM4360.jpg]
