# Supplementary material for: Dual roles of exostosin glycosyltransferase 1 in Zika virus infection
Source: Virulence. 2025 Feb 10;16(1):2458681. doi: 10.1080/21505594.2025.2458681 (PMC11812395; doi:10.1080/21505594.2025.2458681)
Supplement: Supporting_information - clean copy.docx [file KVIR_A_2458681_SM4359.docx]

**Supplementary figure legends**

Figure S1. **The change of autophagy in the HEK-293 and MEF-EXT1-KO cells upon expression of EXT1.** (A) MEF-EXT1-KO cells were transduced by EXT1 containing lentivirus. After 48h, cells were harvested and analyzed by western blot using the antibody against LC3. The representative blots of three experiments were shown. (B) The cell lysates from HEK-293-Vec and HEK-293-EXT1 cells (HEK-293 cells stably overexpressing EXT1) were analyzed by western blot using the antibody against LC3. GAPDH was used as a loading control.

Figure S2. **The effects of EXT1 over-expression on the stability of ZIKV proteins.** The plasmid expressing one of ten ZIKV proteins (capsid protein, prM, E, NS1 NS2A, NS2B, NS3, NS4A, NS4B, and NS5) was co-transfected into HEK-293 cells with or without the plasmid encoding EXT1. The expression of proteins was analyzed by western blots probed with antibodies. GAPDH was used as a loading control. ZIKV E and NS3 were found to be downregulated in the presence of EXT1 expression.
